# Using WeChat as an educational tool in MOOC-based flipped classroom: What can we learn from students’ learning experience?

**DOI:** 10.3389/fpsyg.2022.1098585

**Published:** 2023-01-17

**Authors:** Lanzi Huang, Kai Wang, Shihua Li, Jianwen Guo

**Affiliations:** ^1^Center for Teacher Education Research, Beijing Normal University, Beijing, China; ^2^School of Education, Hunan First Normal University, Changsha, Hunan Province, China; ^3^School of Foreign Languages, Xidian University, Xi'an, Shaanxi Province, China

**Keywords:** MOOCs, flipped classroom, learning experience, higher education, WeChat

## Abstract

Despite its importance, interaction remains limited in MOOC-based flipped classroom (MBFC) Grounded in social learning theory, we proposed an MBFC approach supported by social media to facilitate students’ interaction with peers and learning performance. A quasi-experiment was conducted to compare the MBFC approach (*N* = 58) based on WeChat with the conventional MBFC approach (*N* = 52). The results revealed that the use of WeChat in an MBFC approach led to better performance in terms of watching video lectures and completing online exercises before the class; however, it did not significantly enhance student learning performance compared to the conventional MBFC approach. In addition, the study found that students were moderately satisfied with the MBFC approach supported by WeChat. According to a WeChat interaction quantity and quality analysis, students’ non-substantive postings are much higher than students’ substantive postings in WeChat interaction groups, but students’ contributions to the postings have no significant effect on the final marks. Findings from this study could be of valuable reference for practitioners and researchers who plan to leverage social media tools such as WeChat to support student MOOC learning.

## 1. Introduction

Massive Open Online Courses (MOOCs) are online courses designed for open, unrestricted participation through the Internet (Kaplan and Haenlein, 2016). Since their appearance, many higher institutions have increasingly considered the use of a form of blended learning, commonly known as flipped classroom (FC), where third-party MOOCs are integrated into the curriculum (Fox, 2013; Ebner et al., 2020; Pérez-Sanagustín et al., 2020). In an FC approach, video lectures drawn from a MOOC can be used as a supplement to or a replacement for these traditional face-to-face lectures. MOOC resources play a significant role in students’ learning outside of the classroom (Firmin et al., 2014; Yousef et al., 2015), and many agree that MOOC-based FC approach enhances learning gains in comparison with some traditional approaches (Kloos et al., 2015; Wang et al., 2019; Wang and Zhu, 2019). A study by Zhang (2015) showed that MOOC-based flipped approach was more effective in teaching algorithm than traditional classroom-based approach. Watson et al. (2016) stated that the combination of MOOCs content, technology and a variety of instructor-led activities can help to accomplish teaching objectives effectively.

However, previous research showed that the forum discussion in most MOOC platforms lacked student engagement (Reischer et al., 2017). Less than 5 % of the students interacted with other students in the online forum (Breslow et al., 2013; Rosé et al., 2014). When students are frustrated by the inadequate interpersonal interaction outside of the classroom, the role of information and communication technology in student social interaction becomes a key factor that will affect the nature of student interaction with peers and their instructors (Krause, 2007). Hence, when MOOCs are applied for flipping university courses, a pressing need for students is to have an effective means to discuss and collaborate with their fellow classmates.

The use of social media to support distance education delivery provides new approaches to integrate MOOCs into teaching and learning (McLoughlin and Lee, 2010; Veletsianos and Navarrete, 2012). In fact, social media tools have almost become an indispensable part of people’s daily life, and they have also been increasingly used for educational purposes (Lau, 2017). Social learning theory (Bandura, 2001) advocates the construction of knowledge through social interaction, and students can learn through interaction and communication with their peers. A great number of studies have revealed that using social media tools like Facebook and China’s WeChat can strengthen interaction and collaboration among students and in turn enhance their blended learning experiences (Alammary et al., 2014; Miron and Ravid, 2015; Zhang, 2015). Lin and Hwang (2018) found that the online community-based flipped learning using Facebook could help students become more responsible and autonomous in their learning. The investigation into MOOC-based FC supported by social media tools is relatively new (Wu et al., 2017; Zhang et al., 2018). Therefore, more research is needed to investigate the role of social media tools in the context of MOOC-based FC.

## 2. Literature review

### 2.1. MOOC-based flipped classroom

As high-quality open educational resources, MOOCs provide good support for implementing flipped learning (Wang et al., 2016). The tests and assignments in MOOCs are carefully selected and contain most of the knowledge points, which can provide feedback on students’ inaccurate understanding and performance (Milligan et al., 2013). Currently, MOOCs are being offered by a variety of providers (Veletsianos et al., 2015), including universities, such as Stanford University (Kim and Chung, 2015), the University of Michigan (Severance, 2015), and the University of the Philippines Open University (Bandalaria and Alfonso, 2015), as well as organizations such as the Commonwealth of Learning (Venkataraman and Kanwar, 2015) and the World Bank (Jagannathan, 2015). Generally, anyone with an Internet connection can enroll in MOOCs without admission requirements. Once enrolled, they can access course resources, interact with peers, and share their knowledge with other course participants (Kop, 2011). This educational innovation, which makes higher education more accessible to a massive audience on a global scale, has gained an increasing attention in higher education during the past decade (Carver and Harrison, 2013; Bali, 2014). Researchers have verified the teaching effectiveness based on MOOCs (Wang et al., 2016). An increasing amount of research has been focused on the design of flipped classroom environment, and how this design promotes student engagement and produces better learning outcomes (Geist et al., 2015; Hao, 2016; Chen Hsieh et al., 2017). Some research (e.g., Leis et al., 2015) found that flipped learning is beneficial for students’ language learning. Moreover, de Moura et al. (2021) indicated that he blended mode MOOC can improve the quality problems of MOOCs. Chen and Chen (2021) found that MOOC-based blended learning designs was helpful for providing help and resolving problems.

In a MOOC environment, the course forum plays a major role in acting as the primary communication tool between students with diverse backgrounds (Sharif and Magrill, 2015). Course completers may post more forum posts than non-completers, and the forum posting is considered as an effective measure for student engagement (Kizilcec et al., 2013). Forum discussions usually involve a small number of course participants (Wong et al., 2015). Moreover, forums are ineffective in managing a large number of posts, because their themes are fragmented over many threads. McGuire (2013) observed that forums resulted in complaints from most learners. Suggestions or answers from peers are sometimes incorrect, which may be counter-productive for those seeking answers from the forum. Course participants in Schweizer’s (2013) study acknowledged the benefits of forum discussions for promoting reflection, but also expressed frustration with their overall contribution and claimed them as being unfocused and misleading.

### 2.2. Social media tools: Wechat

Social media tools - often referred as Web 2.0—include a variety of network-related communication technologies (Friedman and Friedman, 2013). These social media tools, such as blogs, wikis and Facebook, enabled users to share images, audios and videos (Hazari et al., 2009). Twitter and Facebook are two popular social media tools in the US and Europe, and the most widely used one in China is WeChat. It is a popular instant messaging (IM) application and social media platform, which enables interactive exchange *via* mobile devices. Until 2018, Tencent’s WeChat monthly active users was 1,057.7 million (Tencent, 2018). Its major features include chatting with friends in live chat sessions, group chat, video calls, voice chat, moments (a timeline where users can “like” or “comment”) and games. Users can receive different services and information by following official WeChat accounts for reading, replying, sharing, and re-posting. Moreover, social media tools are easily used by students with relatively little technological knowledge (Désilets et al., 2005). Due to these desirable features, university students use a variety of social media applications for personal and learning purposes (Cao and Hong, 2011).

### 2.3. Teaching application of social media tools

Social media tools were found to be useful for fostering productive social learning processes (Anderson et al., 2020). Madge et al. (2009) claimed that the use of social media enhances educational access and interaction, and it informally fills the gap in learning between students and teachers. Greenhow and Gleason (2012) suggested that when used in higher education, Twitter may increase the interaction between students and teachers and lead to better engagement. Similary, Fusch (2011) indicated that learning tools are as important as learning objectives. Tools are needed to promote the formation of social groups and the creation of an interactive learning environment and collaborative research. The use of informational technology has created a new way for teachers to communicate with learners (Evans, 2008). Wang et al. (2021) found that combination of MOOCs and social media tools are useful for learners because they provide easier ways to connect with individuals in deep cohesive ways. An online learning environment is more convenient and flexible for learners to arrange their learning plans (Rienties et al., 2012). Heiberger and Harper (2008) claimed that the use of Facebook increased student involvement. Junco (2012) explored the relationship between the type and frequency of Facebook use and student participation. Ajjan and Hartshorne (2008) collected survey data from 136 teachers at a large university in the southeastern United States to investigate teachers’ awareness of the technologies and benefits of adopting Web 2.0 tools in the classroom. Social networking is seen as a useful tool for improving student satisfaction and increasing student interaction with peers. Students showed higher interest and acceptance in blended learning supported by WeChat terminal (Zhao, 2022). Furthermore, Tsai et al. (2017) indicated that the effects of flipped learning and social media tools on students’ computing skills were positive and higher than those who did not use them. However, Alharbi (2015) stressed that students’ being uncomfortable with using social media and an increased workload on the side of the teacher are two drawbacks of this approach. Therefore, the instant messaging function of social media tools deserves further study as it opens up the potential for interactive educational environments (Rambe and Bere, 2013).

This study aims to report our design of MOOC-based flipped classroom (MBFC) incorporating WeChat in a Chinese university. Specifically, our focus is on the effect of MBFC supported by WeChat and learners’ perceptions of it. Four research questions are addressed:

1. How do students in the MOOC-based FC approach supported by WeChat differ in the level of participation as compared to students in the conventional MOOC-based FC approach?
2. Can the MOOC-based FC approach supported by WeChat improve the students’ learning performance in comparison with the conventional MOOC-based FC approach?
3. What are the students’ perceptions of implementing the MOOC-based FC approach supported by WeChat?
4. Is there any difference in WeChat usage data of students in MOOC-based FC approach?

## 3. Methodology

### 3.1. Participants and settings

Participants of this study were students from the 1st and 2nd classes of the advanced mathematics course of Shaanxi Preschool Teachers College. The average age of the participants was 19 years old. The number of students in the two classes was 58 and 52. One class was chosen as the experimental group (EG) and the other one served as the control group (CG). Students in both classes were taught by the same teacher before participating in the study. The teaching week and syllabus of the two classes were the same. An independent *t* test result showed no significant differences (*p* > 0.05) in students’ prior knowledge in the EG (*M* = 39.40) and the CG (*M* = 35.87).

The advanced mathematics course was a four-credit course offered during the winter semester. It was taught twice a week on Monday and Friday with each session lasting for 90 min. The MOOC offered by Tsinghua University was chosen for this study because its teaching schedule fit well with the on-site course. The MOOC included 11 units with five to nine video lectures in each unit, and the length of each video lecture was about 10 min.

For the pre-class learning activity, the students in the EG were randomly assigned to 12 online discussion groups with four to five members in each group. Students used WeChat to check the answers to quizzes, ask questions, and answer questions. For example, students submitted quiz answers in the WeChat group, and members checked answers with each other. Moreover, they were asked to participate in group discussions twice a week and post screenshots of their discussions.

### 3.2. Procedure

**Figure 1:**
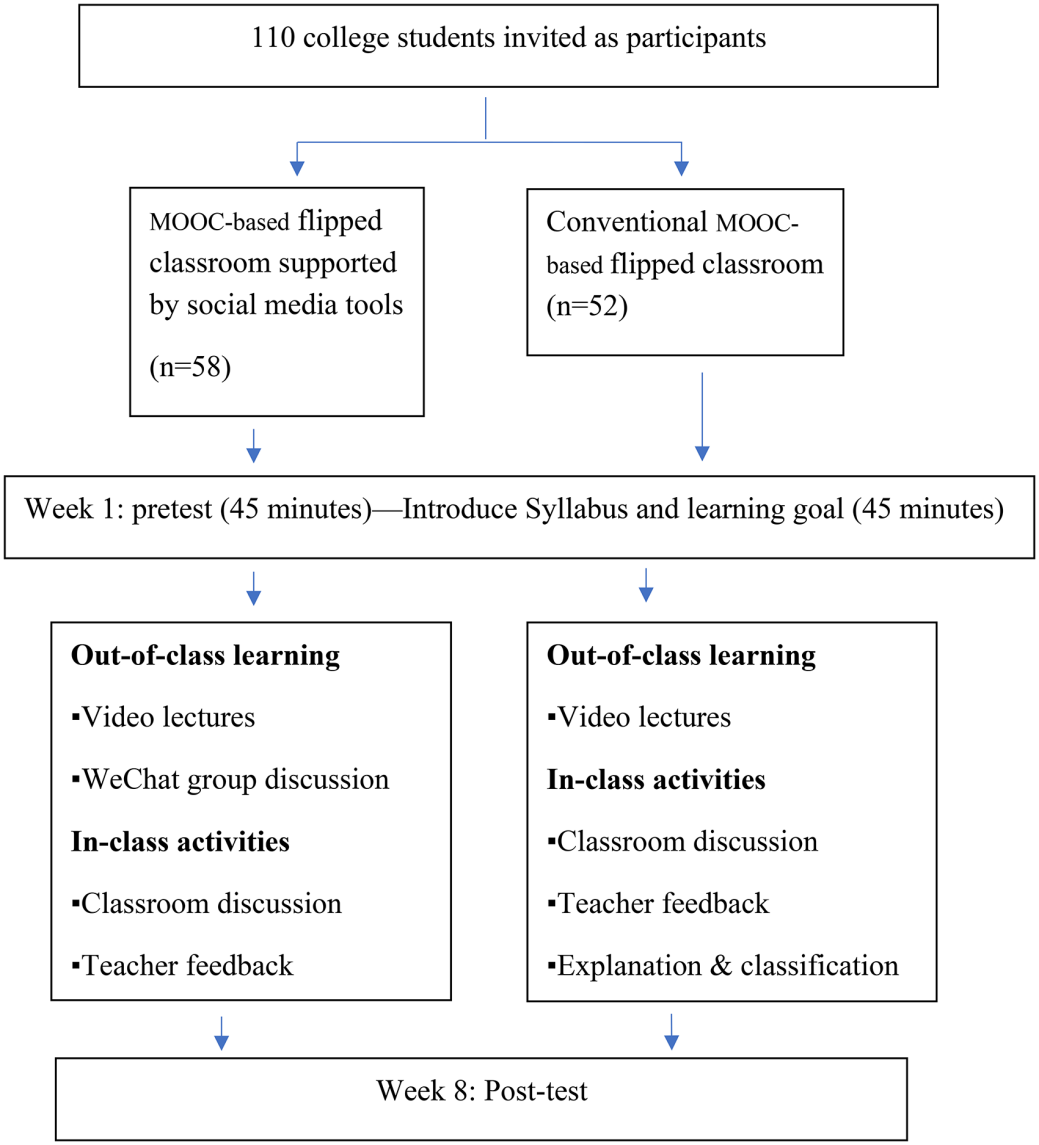
Graphical representation of the research procedure.

This study was conducted over an eight-week period. The schedule of the course is illustrated in Figure 1. During the first week of the course, an introductory session was organized to train students how to access the MOOC and access the video lectures. Over the next 6 weeks, students in both the EG and CG needed to watch two video lectures on the MOOCs platform each week before class and complete 10 quizzes. On top of the MOOC, students in the EG needed to participate in WeChat discussion. During the in-class session, the first 45 min focused on group discussion of questions posted by students in WeChat before class. During this phase, the course instructor would provide assistance to students when they asked for help. During the second period, the course instructor further discussed and clarified the questions to the whole class. In the eighth week, students took the final exam, which served as a post-test.

### 3.3. Instruments

We collected four types of data in this study: students’ participation, students’ learning performance, students’ perceptions, and students’ WeChat usage data.

#### 3.3.1. The students’ participation: Frequency of watching video lectures and completing online exercises

An online survey was used to collect data about student frequency of watching video lectures and completing online quizzes. Students were required to rate their frequency of participation in these two activities as low or high. Students who reported a low frequency received 0 point and those who rated a high frequency received 3 points. The student score for each activity was calculated by using the following formula.

Student activity score = (frequency of students identified the lowest level) * 0 + (frequency of students identified the lower level) * 1 + (frequency of students identified the higher level) * 2 + (frequency of students identified the highest level) * 3.

To illustrate, if a student watched video lectures 11 times or completed online exercises, the student would obtain an activity score of up to 33 and, at worst, an activity weight of 0. Student responses were collected at the end of the experiment. In order to encourage students to answer honestly, the instructor did not have access to the survey data until the grade was submitted.

#### 3.3.2. Learning performance

Prior to the experiment, a student survey was administered to assess their prior knowledge by using a closed test with 20 questions. Each question was worth 5 points. At the end of the course, a post-test was designed to measure learning performance of the two groups. The post-test with a maximum score of 100 points consisted of 12 multiple-choice questions, 4 blank fillings, 5 identification sections, and 3 short answer questions. Two experienced teachers rated students’ learning performance based on the post-test. The inter-rater reliability of the ratings given by the two teachers was 0.738, showing high consistency between their ratings.

#### 3.3.3. Students’ perceptions of the MOOC-based flipped classroom

In order to gage students’ perceptions of the MOOC-based flipped classroom supported by WeChat, a post-task questionnaire survey was conducted. The questionnaire was modified based on the Student Observation Questionnaire (SPIQ) developed by Johnson and Renner (2012). It included 11 items pertaining to students’ perceptions of communication, assessment, and course quality. The questionnaire was administered at the end of the course (see Table 1). The answer to each question used a five-point Likert’s scoring system. The Cronbach’s α values of the subscales were higher than 0.7, respectively, showing an acceptable reliability in internal consistency.

**Table 1:** Students’ perceptions of MOOC-based flipped classroom supported by social media tools.

Items for response	Students’ response	
	Mean	SD
Q1. In the past 6 weeks, I have had a lot of communication with other students.	3.33	0.92
Q2. In the past 6 weeks, I communicated a lot with the teacher.	2.65	0.86
Q3. In the past 6 weeks, I have had to work hard on this course.	3.98	0.75
Q4. In the past 6 weeks, I have learned a lot in this course so far.	3.73	0.84
Q5. In the past 6 weeks, the tasks and projects I have done for this course have dealt with real-life applications and information.	3.38	0.77
Q6. In the past 6 weeks, the availability of course materials, communication, and assessment tools has helped me improve my learning.	3.52	0.72
Q7. In the past 6 weeks, I have applied my out-of-class experiences and learned a lot from practical applications.	3.44	0.87
Q8. In the past 6 weeks, I have explored my own learning strategies.	3.48	0.80
Q9. In the past 6 weeks, I needed technical support to learn this course.	3.25	1.13
Q10. In the past 6 weeks, the availability and access to technical support and resources have helped me improve my learning.	3.50	1.04
Q11. I would choose to take another course like this one.	2.88	1.20
Overall experience	3.37	0.63

#### 3.3.4. Coding scheme for students’ WeChat usage data

Students’ WeChat usage data over 6 weeks were coded and analyzed. The coding scheme developed by Lam et al. (2005) was adopted, which categorized students’ responses into three types: non-substantive, simple substantive and elaborated substantive (see Table 2). When students only get some unconnected information by socializing online, this social interaction is recognized as non-substantive postings. Substantive posting referred to messages that initiated a new discussion thread. In other words, a new topic was explicitly or implicitly presented and then recognized by others (Lee and Tsai, 2011). A detailed answer was “statements that include definitions, examples, comparison, judgments, and predictions” (Hmelo-Silver and Barrows, 2008, p.63). Simple substantive postings referred to those reflecting students’ interaction related to the topics. Postings that generalized and transferred ideas were considered to be elaborated substantive posts.

**Table 2:** Posting model for WeChat platform.

Type of posting	Category	Example
Non-substantive	• Social	Do not forget to watch video lectures on the MOOCs platform
Simple substantive	• Adding new points • Enhancement and clarification of points	Is there any possibility to counteract this problem? Please …
Elaborated substantive	• Making clear contrary statements • Developing complex arguments • Referring to material with a new perspective • Using fresh and different reference material	You can calculate it using the equations and inequalities

The analysis of student WeChat usage data was done collaboratively and iteratively. A student from each group took a screenshot of the discussion and sent it to the research team members on a weekly a basis. At the end of the six-week project, two researchers coded the screenshots according to the coding scheme. For any inconsistent coding, a judge discussed with two researchers until a final agreement was reached.

#### 3.3.5. Data analyses

The data obtained from the questionnaire and the pre-test and post-test scores were quantitatively analyzed using SSPS 19.0. Independent *t*-tests were applied to analyze the differences in the two groups’ pre-test learning performance and students’ participation. Analysis of covariance was then applied to test the variance in post-test learning performance in the two groups. Moreover, the usage data from the WeChat platform were analyzed qualitatively using MAXQDA 12.

## 4. Results

### 4.1. Students’ participation *via* watching video lectures and doing online exercises

Results of the independent *t*-test are presented in Tables 3, 4. The EG watched a greater number of video lectures, by an average of 0.45, which was a significant result (*p* < 0.01). Furthermore, students in the EG completed a greater number of online quizzes than those in the CG, by an average of 0.45, which was also a significant result (*p* < 0.05). Thus, it can be concluded that there were significant differences in the frequency of watching video lectures and completing online exercises between the two groups.

**Table 3:** Results of *t*-test for the frequency of watching video lectures by group.

Class	*N*	*M*	SD	*t*	*p*
Experimental group	58	2.04	0.50	−4.17	0.000**
Control group	52	1.59	0.61		

**p < 0.01.

**Table 4:** Results of *t*-test for the frequency of doing online exercise by group.

Class	*N*	M	SD	*t*	*p*
Experimental group	58	1.85	0.46	−2.55	0.012*
Control group	52	1.60	0.58		

*p < 0.05.

### 4.2. Learning performance

One-way analysis of covariance (ANCOVA) was employed to evaluate the learning performance in the experimental group and the control group by adopting post-test scores as the dependent variable and pre-test scores as the covariate. Table 5 shows the ANCOVA results. No significant difference between the post-test results was observed in both groups (*p* = 0.91 > 0.05) by excluding the impact of the pre-test scores. It can be concluded that there was no significant difference in improving students’ learning performance by each of the two groups.

**Table 5:** The one-way ANCOVA result of the post test of the two groups.

Group	*N*	*M*	SD	*F*	*p*
Experimental group	58	55.16	2.23	7.89	0.91
Control group	52	55.88	1.68		

### 4.3. Analysis of students’ perceptions

The results in Table 1 indicate that overall students in the EG were moderately satisfied with the MOOC-based flipped classroom approach supported by social media tools (*M* = 3.37; SD = 0.63). Very satisfaction response was endorsed for items 2, 6, 7, 8, and 10, and moderate satisfaction response was reported for items 1 and 5. For examples, students reported the specific values of student–student interaction (*M* = 3.33; SD = 0.92) and knowledge acquisition (*M* = 3.52; SD = 0.72). However, students expressed a low level of willingness to continue this new approach of learning (*M* = 2.88; SD = 1.20) because they had to work hard to adapt to this new approach of learning (*M* = 3.98; SD = 0.75). Moreover, it was found that students had negative perceptions of student-teacher interaction (*M* = 2.65; SD = 0.86).

### 4.4. Students’ WeChat usage data

The analyses of WeChat usage data in terms of quantity and quality are summarized in Table 6. In all, there were 569 discussion posts on WeChat platform, and more than 55% of the discussions were non-substantive postings (331 for non-substantive postings, 238 for substantive postings). For the substantive postings, 115 messages were simple substantive postings (i.e., showing agreement or disagreement), and 123 messages were elaborated substantive postings (i.e., involving initiation of a new of discussion). Meanwhile, an average of 9.8 postings per student was recorded after 6 weeks. Each student on average posted less than two messages on the WeChat platform per week.

**Table 6:** Content analysis of postings.

	Postings	Non-substantive postings	Substantive postings	
			Simple substantive postings	Elaborated substantive postings
Total	569	331	115	123
per student (*N* = 58)	9.8	5.7	2.0	2.1
per group (*N* = 12)	47.4	28.2	9.6	10.3

For the quantity of postings, it can be seen that students overall used WeChat platform more actively to socialize than to participate in substantive interaction. Table 7 shows that the total number of postings had no significant direct effect on the final academic performance (*p* > 0.05). The postings were further divided into high and low posting groups (see Table 8), and there was no significant relationship between the high-level postings and the post-test scores (*p* > 0.05). However, it was found that students scoring high in the pre-test had significantly more elaborated substantive postings (*M* = 2.82, SD = 2.65) than those scoring low (*M* = 1.20, SD = 1.38). This suggests that the pre-test score of students had a positive relation with the total number of posts (see Table 9).

**Table 7:** Correlation analysis between postings and academic performance.

	Academic performance	
	Pearson correction	*p*-value
Postings	0.12	0.52

**Table 8:** Post-test for low postings and high postings.

Category	Number of students	Total number of postings	Postings per students	Post-test M (SD)	*p*	*t*
Low	31	87	2.81	60.23 (2.74)	0.73	−0.34
High	27	482	17.85	61.95 (2.95)		

**Table 9:** Prior academic performance and postings.

	Pre-test	Number	Elaborated substantive postings	*p*	*t*
	*M* (SD)		*M* (SD)		
Low	26.60 (1.68)	25	1.20 (1.38)	0.00^**^	−3.00
High	49.09 (1.42)	33	2.82 (2.65)		

**p < 0.01.

## 5. Discussion

With regard to **RQ 1**, the statistical results of this study indicated that students in the EG outperformed the CG with respect to the frequency of watching video lectures and completing online exercises. Our results are consistent with previous studies that identified peer learning communities as a way to promote the development of student engagement (Dodge and Kendall, 2004; Yuan and Kim, 2014). A plausible interpretation of these findings is that sending out reminders in the WeChat platform is a good way to increase the salience of the activity in the learner’s mind. In addition to increasing salience, these reminders can serve as positive reinforcement for active participation and can also trigger intrinsic motivation that guides non-participants to start participating. Doing so may involve social conversations that help students feel recognized, especially in establishing and maintaining relationships (Greenhalgh et al., 2016). Furthermore, the findings support the claim that the affordances of social media tools provide students with an efficient community (Zheng et al., 2016). When students finish watching the video lectures and completing online exercises, they can inform each other about learning experiences, collaborative learning activities, and can also serve as tutors or models for other students, which heighten students’ self-esteem. In turn, this expected achievement will promote students’ positive emotion toward finishing watching video lectures and online exercises. Cultivating a sense of belonging are key or partial goals of digital technology-based networks (Lantz-Andersson et al., 2018).

For **RQ 2**, our results are not consistent with the findings of previous studies (e.g., Lin, 2019) that experience of a learning environment resulted in a change in learning performance. Several reasons may possibly explain this result and would be interesting for future research. First, this is probably related to the fact that the evidence of a relationship between less-controlled technology use (i.e., WeChat platform) and academic performance is still unclear (Rodríguez-Triana et al., 2020). An inherent factor of the use of WeChat platform outside the classroom comes from the anonymity features. Unfortunately, we could not ensure only students in the EG used the WeChat platform. Furthermore, undergraduate students often live on campus in a shared dormitory in Chinese universities, and students can discuss learning problems with their roommates. Face-to-face peer support seems to work better because students can receive quick response to a specific question. Therefore, the WeChat platform may operate only as a secondary channel for students to connect with their classmates. Finally, since students could watch video lectures at their own pace, those who start late or fall behind might not keep up with the discussion and postings on the WeChat platform. Moreover, students may wish to receive trustworthy responses. This means that in general students expect a response from “an expert” rather than an uncertain response from their peers. In addition, using social media tools in the learning process might lead to misunderstanding, reduced knowledge sharing, and reduced creative thinking (Hrastinski and Aghaee, 2012).

Regarding **RQ 3**, Our results also indicated there were no significant differences between students’ learning experience in the flipped classroom using or not using a social media tool. Jones et al. (2010) found that students’ perceptions of technology use in social life and the learning space varied widely. More than 70% of students reported that they rarely used social media tools for learning despite having social network accounts. This suggests that the WeChat platform should be understood as a more socially orientated platform rather than one for problem-solving. Furthermore, although students in the MOOC-based flipped classroom supported by a social media tool completed more video lectures and online exercises, this does not mean that they understood the content of the course better. In addition, most of the students in both groups were unfamiliar with this new learning format, in which students needed to cooperate with other students. Therefore, it is difficult to see the difference in students’ experience in a short term.

Turning to **RQ 4**, the results of this study showed that students were more willing to socialize than to learn through the WeChat platform. The finding supports the that of Sun et al. (2018), which found that social media tools may increase the overall quantity of interactions, but may not result in high levels of knowledge construction. The results are also probably related to the fact that students may not get actively involved in using the WeChat platform if there are no clear expectations or no rewards (e.g., grades) given to them for their contribution. This corresponds to the finding of Dennen’s (2005) study that students’ contributions were plagued by unclear teacher expectations because students did not know how much they contributed or what their postings should look like. As a result, students may not post any messages throughout the semester if no grade is attached to the postings in online discussions. Other studies also reported that deep learning is less likely to occur in online discussion forums—similar to WeChat platform in our study—than in face-to-face format of learning (McCrory et al., 2008), and students prefer to use other media with equivalent capabilities (Ezeah, 2014).

Meanwhile, our result indicated that there was no significant correlation between learning performance and the use of social media tool. This is inconsistent with the studies of Junco et al. (2011) and Stollak et al. (2011) which found that the use of social media tools was significantly improved undergraduate students’ engagement and GPA. The results can be explained by the fact that students posted a much high proportion of less task-focused threads on the WeChat platform. Prior research also reported that posting unprofessional content was common in such environments (Chretien et al., 2009). In another study, Junco (2012) found that time spent on the social media tool “Facebook” was negatively correlated with the GPAs of college students since it had little to do with the time to prepare for the courses. Moreover, participating in the online discussion increased students’ workload when they had to attend the face-to-face classes later (Seethamraju, 2014). Another possible explanation could be that the results of the final exam score did not fully reflect the use of social media tool, such as knowledge construction (Noroozi et al., 2013) and critical thinking skills (Zhang et al., 2007).

Furthermore, different from the prior studies that students achieving the highest final marks has the highest frequency of postings, our findings do not reveal differences in final marks between high and low participation groups (Xia et al., 2013; Koole et al., 2014). This can be interpreted as the fact that some students who read WeChat posting regularly did not make postings (Mustafaraj and Bu, 2015). This reading-only form of participation in online discussion forums is “latent.” The participants of this study were freshmen who just started college and had not developed a close rapport with their classmates. Thus, they might not feel free to express their ideas because of shyness. In spite of remaining invisible, some students would engage in the discussion when they found it useful to their learning. Other studies (e.g., Webb et al., 2004) also reported that both active participation and passive participation may benefit online users. The results can also be explained by the fact that irrelevant postings by students and insufficient moderation by the teacher did not improve the learning of high participation groups (Chen et al., 2011).

However, our result points out that students who scored high in the pre-test were significantly different from those who scored low in terms of the number of elaborated substantive postings. This result is consistence with the previous findings of Green et al. (2014) that more academically capable students posted more regularly on online discussion. This suggests that prior academic performance in this study might be considered a key indicator of the quality of postings. Students with relatively high academic ability were more motivated to learn the course (Green and Hughes, 2013), and posted in the WeChat group discussions. Conversely, when students with low academic performance are not able to solve problems they encounter, they may decide not to seek help for personal reasons (e.g., embarrassment or fear of appearing incompetent), or they may perceive help seeking as a form of non-elaborated help, especially when non-elaborated help leads to the student’s requests being ignored.

## 6. Conclusion and implications

This study proposed a MOOC-based flipped classroom approach supported by a social media tool. Moreover, a quasi-experiment was conducted to evaluate the learning effectiveness of the proposed approach. The results showed that the proposed approach significantly improved the students’ online participation in watching video lectures and completing online exercises but not students’ final grades. Furthermore, the students were moderately satisfied with the proposed approach. In addition, this study combined higher-level conceptions (e.g., elaborated substantive postings) with frequency counts to gain an in-depth understanding of how a social media tool impacts the MOOC-based flipped classroom approach.

Our findings have some implications for practitioners. When designing online group discussions by using social media tools in the context of MOOC-based flipped learning, it is advisable for teachers to consider the relationship between identified maladaptive factors and effectiveness, develop appropriate strategies to support students’ online discussions, and ultimately guide their success. Moderation is considered one important design element essential for productive conversations to occur (Andrews-Larson et al., 2017). Meanwhile, moderators (i.e., teachers) are needed to structure the use social media tools in learning, for example by organizing chats with questions related to a common theme (Greenhalgh et al., 2020).

Moreover, students were found to express a low level of willingness to continue this new approach because they had to work hard to adapt to it. Therefore, we advise careful consideration of the frequency and mode of the new approach, as it could make students dissatisfied when they see it as a burden. We suggest presenting a social media tool support in a scaffolding strategy, in which the MOOC-based flipped learning instruction tips build on each other and are gradually reduced to encourage the students to internalize the social media tools strategies instructed. As the use of social media tools by college students and teachers continues to grow, it is hoped that this research will lead to further comparative studies about WeChat and similar tools in order to assess the better use of emerging technologies in an educational environment.

Although this study is relevant to practice and research, there are some limitations. Firstly, this study was conducted using a small sample of the entire student population. Future research should explore students from across different disciplines and provide additional evidence. Secondly, the duration of experiment constitutes a constraint on the results. Future research could consider verify the results in a design where a social media tool is implemented in a MOOC-based flipped classroom for a longer period. Finally, the result does not fully reflect the impact of the proposed approach on student learning performance. Therefore, to address this issue, a further experiment should be conducted to investigate the student learning performance of an advanced mathematics course among a conventional flipped classroom, a MOOC-based flipped classroom, and a MOOC-based flipped classroom supported by social media tools and further investigate the effect of the social media tools on student learning performance in the flipped classroom.

## Data availability statement

The original contributions presented in the study are included in the article/supplementary material, further inquiries can be directed to the corresponding author.

## Ethics statement

Ethical review and approval was not required for the study on human participants in accordance with the local legislation and institutional requirements. Written informed consent for participation was not required for this study in accordance with the national legislation and the institutional requirements.

## Author contributions

KW and SL conceived and designed the study. KW performed the quasi-experiment and collected data. After that, KW wrote the paper. LH and JG reviewed and edited the manuscript. All authors contributed to the article and approved the submitted version.

## Conflict of interest

The authors declare that the research was conducted in the absence of any commercial or financial relationships that could be construed as a potential conflict of interest.

## Publisher’s note

All claims expressed in this article are solely those of the authors and do not necessarily represent those of their affiliated organizations, or those of the publisher, the editors and the reviewers. Any product that may be evaluated in this article, or claim that may be made by its manufacturer, is not guaranteed or endorsed by the publisher.
